# Extracting information from noisy data: strain mapping during dynamic in situ SEM experiments

**DOI:** 10.1557/s43578-020-00041-0

**Published:** 2021-01-19

**Authors:** M. Alfreider, M. Meindlhumer, V. Maier-Kiener, A. Hohenwarter, D. Kiener

**Affiliations:** 1 Chair of Materials Physics, Department of Materials Science, Montanuniversität Leoben, 8700 Leoben, Austria; 2 Christian Doppler Laboratory for Advanced Synthesis of Novel Multifunctional Coatings at the Department of Materials Science, Montanuniversität Leoben, 8700 Leoben, Austria; 3 Chair of Physical Metallurgy and Metallic Materials, Department of Materials Science, Montanuniversität Leoben, 8700 Leoben, Austria

## Abstract

**Abstract:**

Micromechanical testing techniques can reveal a variety of characteristics in materials that are otherwise impossible to address. However, unlike to macroscopic testing, these miniaturized experiments are more challenging to realize and analyze, as loading and boundary conditions can often not be controlled to the same extent as in standardized macroscopic tests. Hence, exploiting all possible information from such an experiment seems utmost desirable. In the present work, we utilize dynamic in situ microtensile testing of a nanocrystalline equiatomic CoCrFeMnNi high entropy alloy in conjunction with initial feature tracking to obtain a continuous two-dimensional strain field. This enables an evaluation of true stress–strain data as well as of the Poisson’s ratio and allows to study localization of plastic deformation for the specimen. We demonstrate that the presented image correlation method allows for an additional gain of information in these sophisticated experiments over commercial tools and can serve as a starting point to study deformation states exhibiting more complex strain fields.

**Graphic abstract:**

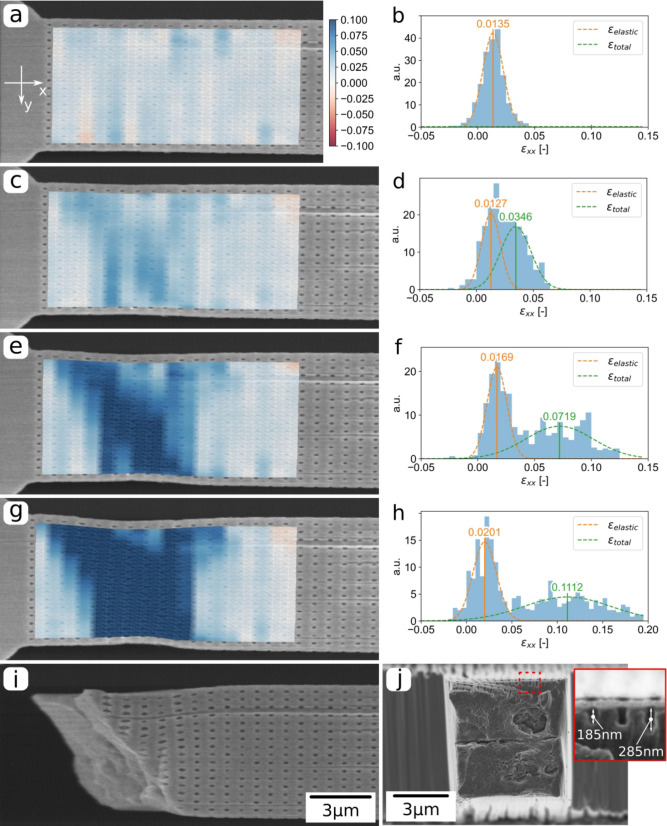

## Introduction

In situ micromechanical testing techniques inside scanning electron microscopes (SEMs) are highly utilized tools to study deformation and fracture characteristics of a wide range of materials at scales that were previously unachievable [1, 2]. Thereby, they provide the possibility to investigate, for example, size effects [3], irradiation layers [4] or microstructural features [5] at their native scale. While indentation based techniques, such as nanoindentation or micropillar compression, are by far the most common methods, tensile testing in such in situ setups has been conducted less frequently, as the experimental effort is considerably higher [6–8]. Independent of whether a push-to-pull geometry [8–12] or a gripping geometry [6, 13–17] is employed, the sample preparation is more tedious and the time required to conduct an experiment is distinctly longer compared to a single nanoindentation or microcompression test. Therefore, it is desirable to extract as much information as possible from such an individual tensile experiment. As these investigations are typically conducted in situ within a microscope, it is reasonable to incorporate the acquired images during the experiment as additional information into the analysis and thereby reaching out into the vast research field of digital image correlation (DIC) [18–20]. Various groups have been conducting SEM-DIC on different material systems, with the main focus on high resolution and small strains [19, 21–25]. The major challenge in SEM-DIC is taking into account the individual distortions based on temporal and spatial drift, as well as deviations in illumination based on the scanning nature of image acquisition in SEMs. Various methods to reduce these errors have been presented in the literature, such as iterative removal of drift and spatial distortion [21–24] or even adjusting the scan generation unit to ensure a more uniform illumination [25]. However, all of these approaches require high resolution SEM images with a low signal to noise ratio, which is synonymous with slow scanning speeds and acquisition times of several minutes per frame, thus a static image. In contrast, in miniaturized experiments a continuous loading is desired to ensure a constant strain rate as well as to neglect any stress-relaxation phenomena that could occur during a static holding segment. Therefore, it is necessary to drastically reduce the time for recording of each individual frame to ensure a quasi-continuous image acquisition during the experiment. This consequently results in lower image quality and higher image noise, rendering the classical DIC approach (cross-correlation of subset windows) [18] nearly impossible. Hence, it is required to employ a different approach, based on tracking of individual features that are easily visible even on lower quality images. In the present work, we utilize point features introduced by focussed ion beam milling as spots to track during the deformation to circumvent the prerequisites for classical DIC [18, 26, 27]. This enables calculation of the strain field on the specimen based on quadrilateral elements and allows for more detailed evaluation of stress–strain data. Specifically, for micro-tensile experiments, where machine compliance is known to be an issue, it improves the measurement of strain along the tensile axis. We complement this investigation by spherical nanoindentation as an alternative technique for local flow curve measurement to highlight that the DIC approach offers additional information in terms of strain. Furthermore, the strain measurement perpendicular to the tensile axis allows for an estimate of true stress, even after necking, as well as Poisson’s ratio, which would not be accessible by nanoindentation.

## Point tracking and image correlation

**Figure 1:**
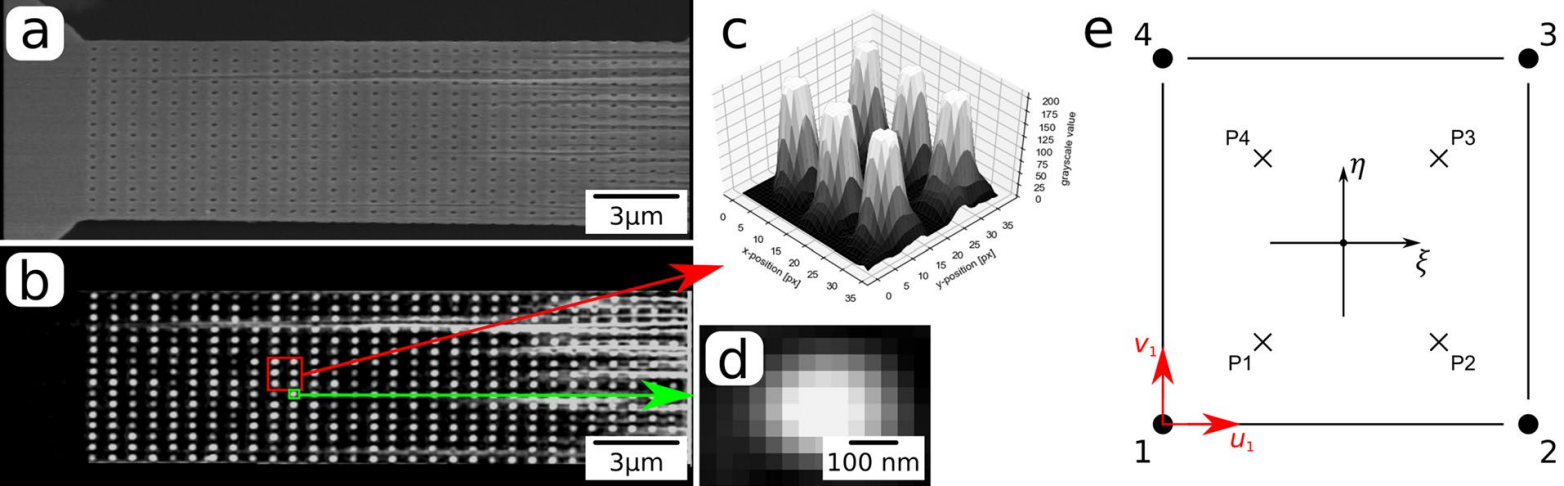
(a) Raw and (a) processed image of the specimen gauge section before testing, and (c) a 3D plot of the grayscale values of a subset indicated in (b), as well as (d) a zoomed image, showing the local maxima used for point tracking. (e) Quadrilateral element for strain evaluation in normalized *ξ*,*η*-space. The points 1 to 4 correspond to experimentally tracked points with respective displacements *u*_*i*_,*v*_*i*_ in *x*,*y*-space. P1 to P4 are the chosen points for strain evaluation.

To address the deformation of the specimen, it is necessary to track the visual movement of the individual points on its surface. Therefore, the images were pre-processed using ImageJ 1.52s, applying the implemented edge detection algorithm followed by a Gaussian filter with a kernel size of 2 px, to achieve greyscale maxima on each tracking feature (point), respectively. The resulting raw and processed greyscale images of the specimen before testing are shown in Fig. 1a, b. In the processed case (Fig. 1b) the edges of the specimen were further removed, to minimize any error during computation, as only the point features should be tracked. The centres of the points were evaluated as the peak of a two-dimensional Gaussian fit for each individual point at every tenth image, which resulted in a good trade-off between computational effort and temporal resolution. The two-dimensional Gaussian form was assumed based on the local circularity (Fig. 1d) and peak shaped structure (in greyscale space), shown in a three-dimensional plot in Fig. 1c for an inset of the processed image. The consecutive point positions were determined for each subsequent frame by allowing only small incremental displacement of ± 7 px for each step. This assures correct tracking of the respective point and avoids false influences by neighbouring ones. Furthermore, during routine development, each incremental step was checked manually by plotting the points on top of the raw images to ensure exclusion of any obvious non-physical outliers. The displacements were then calculated by subtraction of the respective initial reference positions on the undeformed specimen.

### Strain evaluation

Evaluating the local strain in the specimen is not straightforward, as it is a function of the individual displacements surrounding the investigated location. To address this issue, an approach usually known from finite element analysis (FEA) is used in the form that individual quadrilateral elements are introduced. These are inscribed in the area between four connected points and allow for a description of displacements inside the element by so called shape functions (*N*_1_, *N*_2_, *N*_3_, *N*_4_) [28]:

1 $$\begin{gathered} N_{1} = \frac{1}{4}\left( {1 - \xi } \right)\left( {1 - \eta } \right) \hfill \\ N_{2} = \frac{1}{4}\left( {1 + \xi } \right)\left( {1 - \eta } \right); \hfill \\ N_{3} = \frac{1}{4}\left( {1 + \xi } \right)\left( {1 + \eta } \right); \hfill \\ N_{4} = \frac{1}{4}\left( {1 - \xi } \right)\left( {1 + \eta } \right); \hfill \\ \end{gathered}$$

where *ξ* and *η* are coordinates in normalized space (from − 1 to 1) as shown in Fig. 1e. Therein the points 1 to 4 correspond to the tracked points during the experiment, with respective displacements *u*_*i*_ in *x*-direction and *v*_*i*_ in *y*-direction. Based on this formalism, the displacements and resulting strains can be calculated in the entire element. However, as the shape functions are linear and the graphical strain visualization is consequently not influenced to a noticeable extent by interpolation between the points, we settled on the four evaluation points P1 to P4 at variations of − 0.5 and 0.5 in the *ξ,η*-space as shown in Fig. 1e.

To acquire strains in two dimensions, it is first necessary to obtain the gradients of the displacements with respect to the *x,y*-coordinates, *i.e.* $\frac{\partial u}{{\partial x}};\frac{\partial v}{{\partial y}};\frac{\partial u}{{\partial y}};\frac{\partial v}{{\partial x}}$ for the respective points. The computation of these gradients is given by the sum of the gradients of the shape functions with respect to the *x,y*-coordinates times the respective displacement, as *e.g.* [28]:

2 $$\frac{\partial u}{{\partial x}} = \mathop \sum \limits_{i = 1}^{4} \frac{{\partial N_{i} }}{\partial x} u_{i}$$

For the sake of conciseness, only the gradient $\frac{\partial u}{{\partial x}}$ is shown, while the others are constructed correspondingly. The main challenge here is to find the shape function gradients in real space, as they are described in *ξ,η*-space. However, their gradients with respect to the *ξ,η*-coordinates can be evaluated straight forward by hand for the given points (P1–P4). This allows utilizing the form [28]:

3 $$\left[ {\begin{array}{*{20}c} {\frac{{\partial N_{i} }}{\partial x}} \\ {\frac{{\partial N_{i} }}{\partial y}} \\ \end{array} } \right] = \left[ {\begin{array}{*{20}c} {\frac{\partial \xi }{{\partial x}}} & {\frac{\partial \eta }{{\partial x}}} \\ {\frac{\partial \xi }{{\partial y}}} & {\frac{\partial \eta }{{\partial y}}} \\ \end{array} } \right]\left[ {\begin{array}{*{20}c} {\frac{{\partial N_{i} }}{\partial \xi }} \\ {\frac{{\partial N_{i} }}{\partial \eta }} \\ \end{array} } \right] = {\varvec{J}}^{ - 1} \left[ {\begin{array}{*{20}c} {\frac{{\partial N_{i} }}{\partial \xi }} \\ {\frac{{\partial N_{i} }}{\partial \eta }} \\ \end{array} } \right]$$

where ***J***^−1^ is the inverse of the Jacobian of the *x,y*-system with respect to the *ξ,η*-coordinates. There, the respective gradients can be constructed as:

4 $$\frac{\partial x}{{\partial \xi }} = \mathop \sum \limits_{i = 1}^{4} \frac{{\partial N_{i} }}{\partial \xi } x_{i}$$

where *x*_i_ are the respective *x*-positions of the 4 points in real space. Again only a single gradient expression is shown for conciseness.

Using Eqs. (2) to (4) allows for the construction of a two-dimensional deformation gradient tensor ***F*** to link the deformed and reference state as [28]:

5 $${\varvec{F}} = \left[ {\begin{array}{*{20}c} {1 + \frac{\partial u}{{\partial x}}} & {\frac{\partial u}{{\partial y}}} \\ {\frac{\partial v}{{\partial x}}} & {1 + \frac{\partial v}{{\partial y}}} \\ \end{array} } \right]$$

to finally calculate a Green–Lagrange strain tensor ***E*** as [28]:

6 $$E = \frac{1}{2}\left( {{\varvec{F}}^{{\text{T}}} {\varvec{F}} - {\varvec{I}}} \right) = \left[ {\begin{array}{*{20}c} {\varepsilon_{xx} } & {\varepsilon_{xy} } \\ {\varepsilon_{xy} } & {\varepsilon_{yy} } \\ \end{array} } \right]$$

which is composed of the respective strains in *x*-direction (*ε*_xx_) and y-direction (*ε*_yy_) as well as the shear components (*ε*_xy_) and has the advantage of taking large displacements as well as rotations into account.

## Results

### Specimen geometry and measured load–displacement response

**Figure 2:**
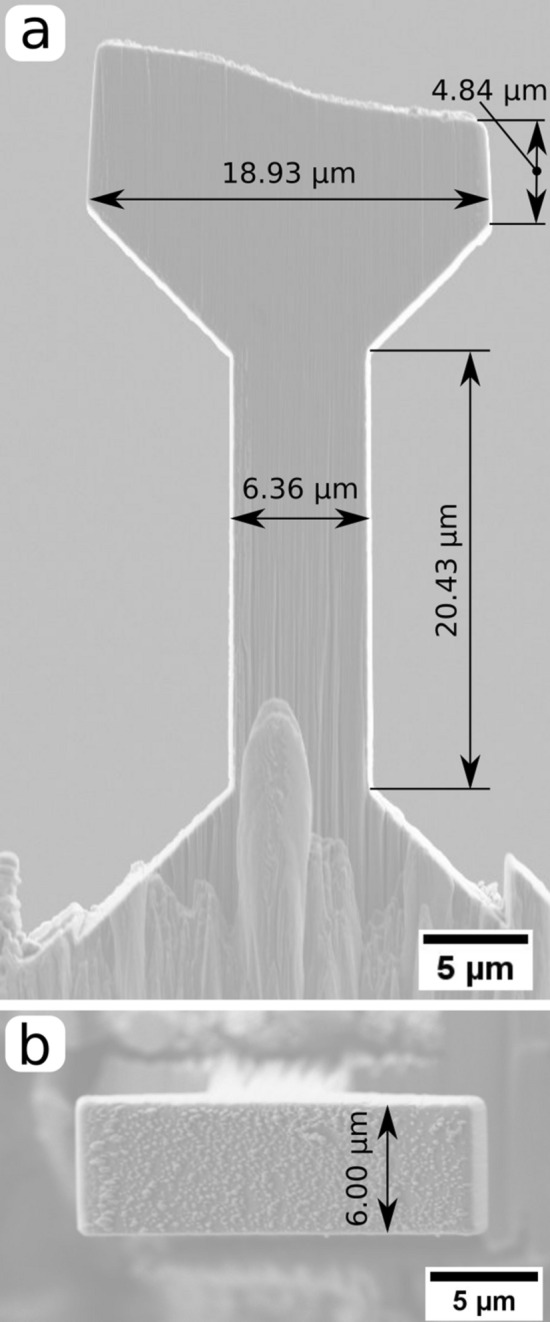
SEM images of the micro-tensile specimen geometry depicted from (a) the backside and (b) the top.

**Figure 3:**
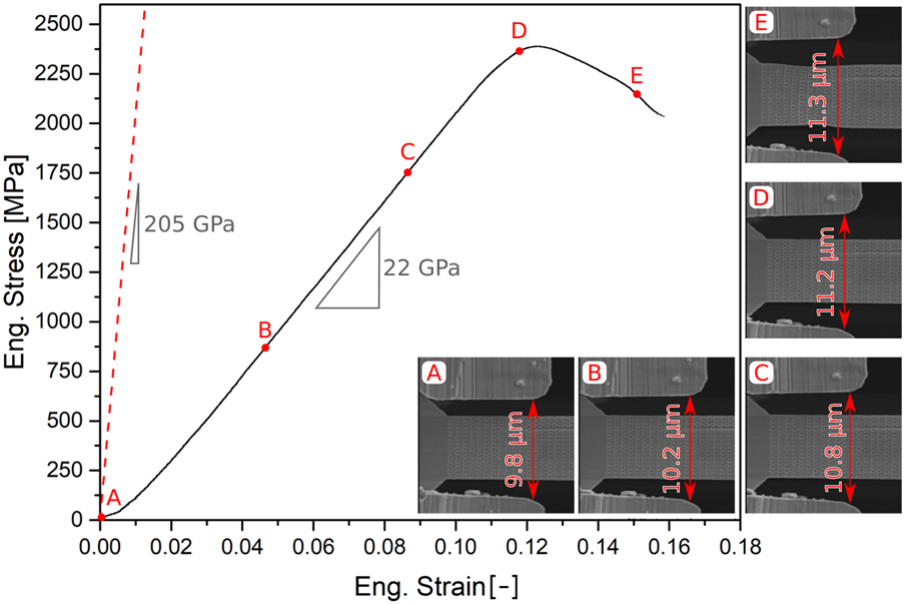
Engineering stress–strain data calculated from raw load and displacement, showing an uncorrected apparent loading modulus of 22 GPa. For comparison the nanoindentation modulus of 205 GPa is added. Furthermore, insets depicting the increasing gripper width during pulling are shown at different points throughout loading (a–e).

The testing geometry is shown in Fig. 2, with the initial specimen dimensions of length *l*_0_ = 20.43 µm, width *w*_0_ = 6.36 µm and thickness *t*_0_ = 6.00 µm, respectively. The feature in front of the specimen is residual material from the FIB process, which is not in contact with the specimen. As can be seen from the view down the length axis of the specimen in Fig. 2b, the taper was fully compensated, resulting in a cross sectional area of *A*_0_ = 38.16 µm^2^. Utilizing these measurements in connection with the gathered load–displacement data allows to evaluate the engineering stress–strain response as shown in Fig. 3. There, a non-constant slope up to ~ 100 MPa is evident, followed by a linear loading regime up to ~ 2100 MPa, before an ultimate tensile stress (UTS) of 2387 MPa is reached. Thereafter, the slope decreases until failure. The initial increase in slope can be attributed to the establishment of a solid contact before elastic loading of the sample takes place. All in all, the specimen shows continuous deformation without sudden load drops, suggesting that a representative bulk response is measured. Nevertheless, enumerating the slope of the linear elastic region, results in a measured apparent modulus of only 22 GPa, which is an order of magnitude lower than the modulus measured by nanoindentation investigations on the exact same material (*E*_indentation_ = 205 GPa) [29] or the modulus measured by resonance frequency measurements on bulk material (*E*_resonance_ = 203 GPa) [30]. The indentation modulus slope is indicated for comparison in Fig. 3. This suggests that a non-negligible amount of machine and contact compliance is present, resulting in a very challenging condition to measure stress–strain response exclusively from load–displacement data [31].

One issue of the high machine compliance is evident from the in situ images, where a strong elastic distortion of the gripper during the experiment is visible. The insets in Fig. 3a–e show the open width of the gripper upon subsequently increasing loads during the experiment. The gripper bends open by a maximum of 1.5 µm (from 9.8 to 11.3 µm), which gives rise to sliding down of the specimen and therefore an increased machine compliance. Notably, this is an extreme situation, but serves well to depict the generally persistent issue, in particular for strong materials. To counteract this and still use the displacement of the indenter, it would be necessary to know the inherent machine compliance and contact compliance, which is not only dependent on the gripper but also on the shape and inherent material properties (*e.g.* flow stress) of the base material under investigation. One way to address this issue would be to simulate the full contact situation including the gripper, *i.e.* utilizing finite element analysis (FEA), to subtract the calculated machine compliance for each data point, which would only be possible in an iterative manner as it requires knowledge of all unknown material parameters. With this in mind, an independent measurement of specimen strain from digital image correlation techniques becomes even more appealing.

### Results from DIC strain measurement

The evaluation of strain was conducted on the leftmost part of the specimen gauge section, as in the rightmost area curtaining artefacts from FIB processing inhibited the point tracking on processed images, through obscuring the individual points by line features (see Fig. 1b). However, the sample showed obvious plastic deformation, necking, and final failure in the centre of the evaluated area of interest, while the lower part of the specimen only deformed elastically. Thus, a total of 288 individual points was tracked, which resulted in 255 quadrilateral elements with 1020 evaluation points.

**Figure 4:**
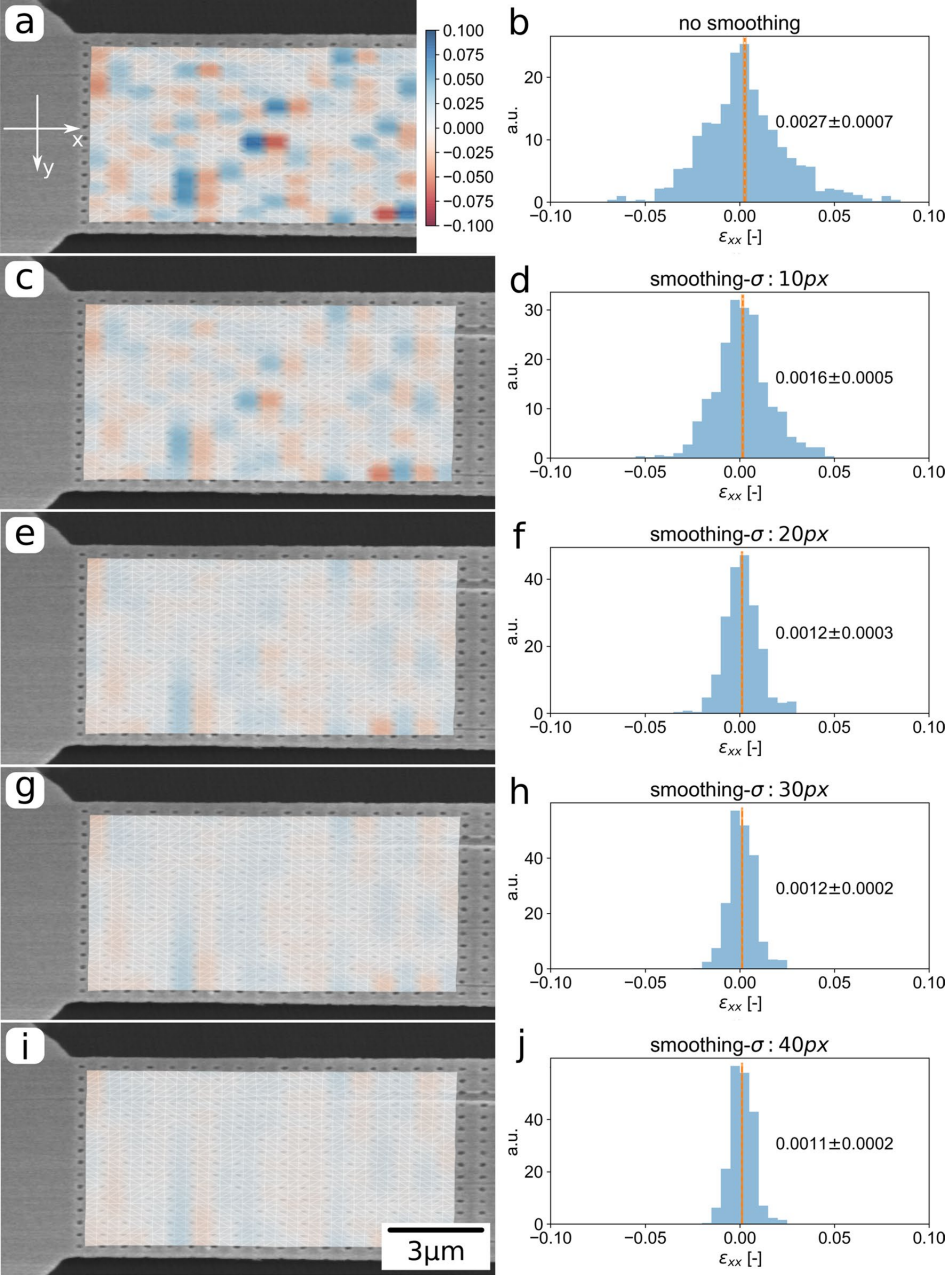
Strain in *x*-direction of the first evaluated image during elastic loading at 432 MPa (a) as calculated from the raw data and (c), (e), (g), (i) for different radii *σ* of the smoothing algorithm. The colour code and micron bar are valid for all images. The histograms in (b), (d), (f), (h), (j) depict the corresponding distribution of strain as well as mean and standard error, respectively.

The calculated strain in *x*-direction (loading axis of the specimen) of the first evaluated image in the elastic regime at an engineering stress of 432 MPa is shown in Fig. 4a, where blue corresponds to tensile and red to compressive strains. The colour palette is arranged symmetrically around zero with maxima at ± 0.1. From Fig. 4a it is evident that the calculated data exhibits a significant contribution from stochastic behaviour, which is unphysical and results from the measurement deviations of the individual points. In Fig. 4b a histogram of all data points is shown to quantify this stochastic spread. There, the bell shaped form of the data suggests a normal distribution with a mean value of 0.27% and a standard error of 0.07%, which seems reasonable for the elastic regime (432 MPa).

To reduce stochastic changes in strain, it is necessary to decrease steep changes in gradients of displacement from one point to another, while still keeping actual physical displacement intact. This can be achieved using smoothing algorithms. Here, we apply the idea of smoothing the displacement (*u,v*) field using a 2-dimensional Gaussian kernel with varying radius *σ* under the assumption that no distinct deformation features, *e.g.* shear band or glide steps occur, as:

7 $$\begin{gathered} u_{{{\text{smooth}}}} \left( {x,y} \right) = \int {\int\limits_{ - \infty }^{\infty } {u\left( {x,y} \right)\frac{1}{{2\pi \sigma^{2} }}e^{{ - \frac{{x^{2} + y^{2} }}{{\sigma^{2} }}}} {\text{d}}x {\text{d}}y} } \hfill \\ v_{{{\text{smooth}}}} \left( {x,y} \right) = \int {\int\limits_{ - \infty }^{\infty } {v\left( {x,y} \right)\frac{1}{{2\pi \sigma^{2} }}e^{{ - \frac{{x^{2} + y^{2} }}{{\sigma^{2} }}}} {\text{d}}x {\text{d}}y} } \hfill \\ \end{gathered}$$

Implementation of Eq. (7) is accomplished numerically by exchanging the double integral by a double sum and calculating the discrete values at every pixel up to a maximum radius of 100 px around each point, respectively.

**Figure 5:**
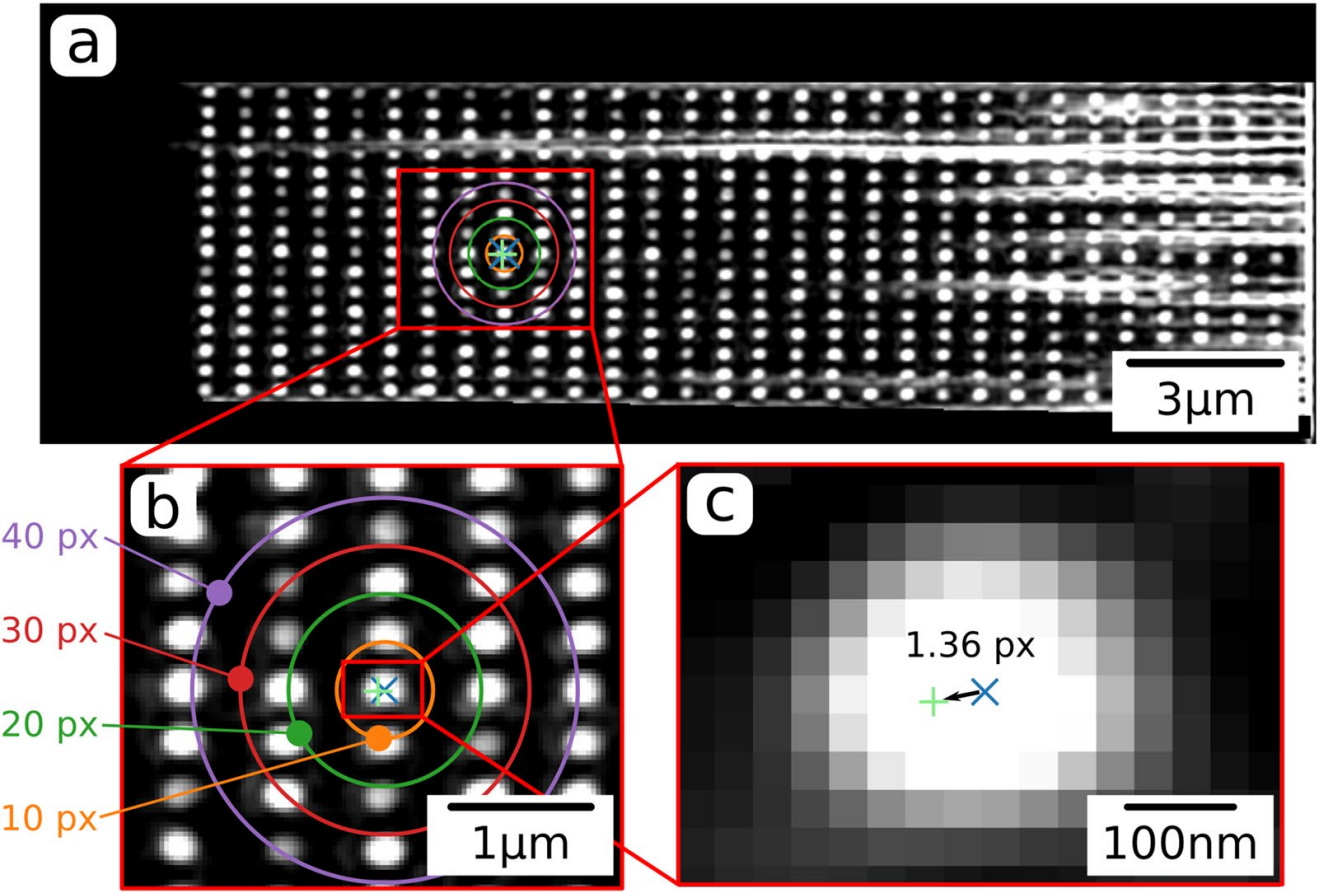
(a) The post-processed image of the sample at 432 MPa with (b) the Gaussian kernel radii *σ* in 10 px steps up to 40 px and (c) the point with the largest shift of 1.36 px after the smoothing algorithm using a radius of 40 px.

Figure 5 shows the post-processed image at 432 MPa with a focus on the point with the largest absolute shift using the proposed algorithm. The zoomed detail (Fig. 5b) depicts different radii of the Gaussian kernel in 10 px steps and the enlarged detail (Fig. 5c) shows the corresponding point positions in the deformed configuration as raw data (blue x) and after the algorithm with a kernel radius of 40 px (green+). This corresponds to the point with the highest shift of 1.36 px, and it is evident that it is still well inside the tracking feature. Strain-fields and distributions with different kernel radii are shown in Fig. 4 in correspondence with the one obtained from the raw data. Evidently, the stochastic nature of the fields decreases with increasing kernel radius. Furthermore, the strain distributions still depict a bell-shaped characteristic, suggesting that the algorithm did not change the normal distribution of strains, while considerably decreasing the width of it. However, it is also evident that the mean values decrease with increasing kernel radius, which is a consequence of the smoothed displacement field. As the strain is a function of the first order gradients of the displacement field and the squares thereof, smoother displacement changes lead to less steep gradients and therefore a lower mean strain as the absolute value of the squared terms decrease due to shorter distribution tails. This, furthermore causes over-smoothing and consequently undesired removal of actual features.

**Figure 6:**
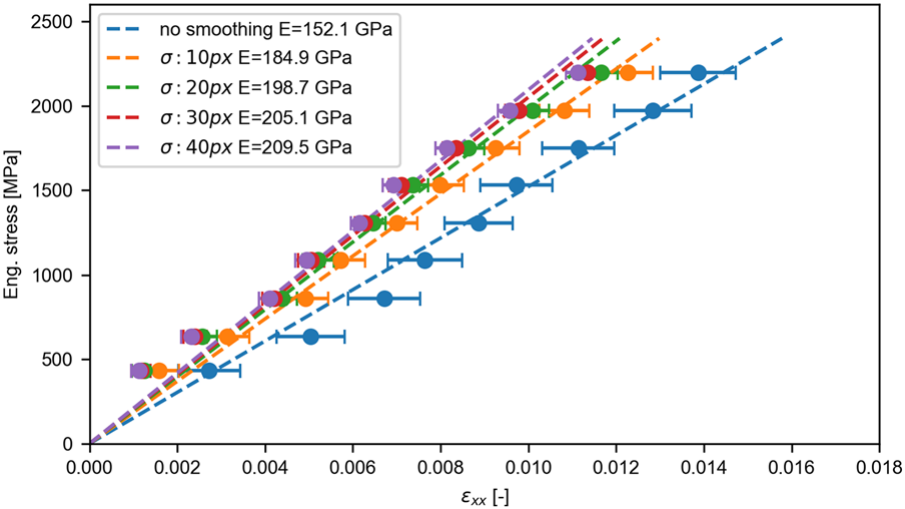
Engineering stress—mean strain data in the elastic regime for different kernel radii up to 40 px, as well as derived Young’s moduli indicated in the data legend.

To identify the accurate kernel radius for adequate evaluation, mean strains and corresponding standard errors were calculated for 9 frames in the elastic regime and kernel radii up to 40 px, and were subsequently used to calculate the Young’s moduli *E*, by linear regression with a forced intercept at the origin, respectively. The summarized data is shown in Fig. 6, where the error bars correspond to the standard error of the mean for each individual data point. Based on this data we conclude that a kernel radius of 40 px overestimates the Young’s modulus from indentation data (205 GPa), while a kernel radius of 30 px results in a rather good agreement with said data. Therefore, the following evaluation will be based on data processed with a kernel radius of 30 px.

**Figure 7:**
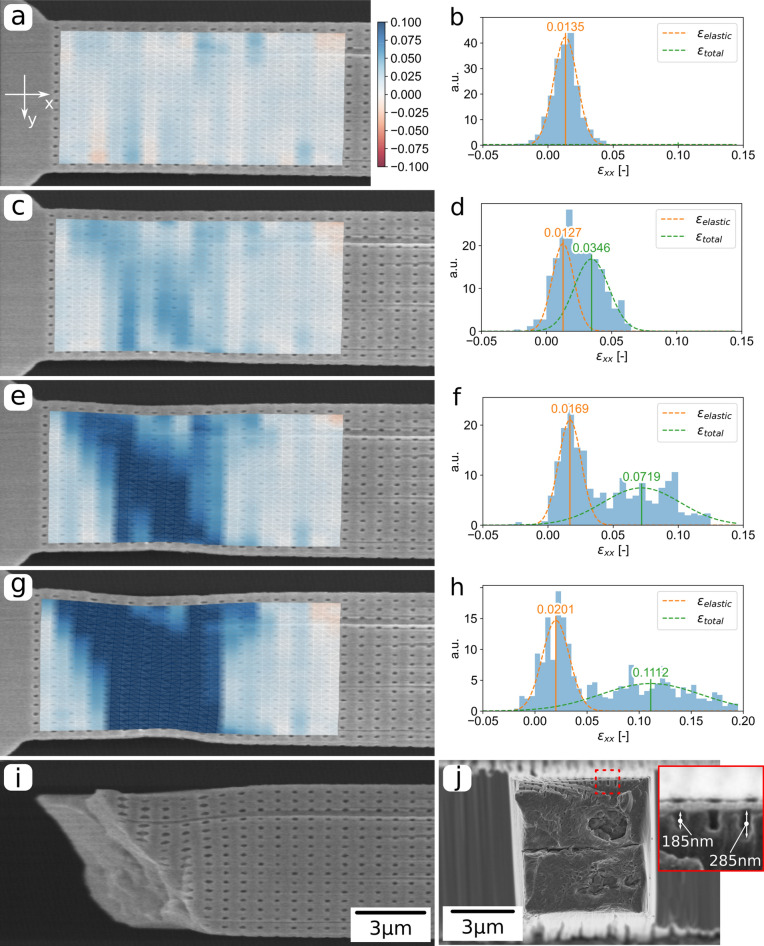
Strain fields in *x*-direction for various loading steps: (a) before evident deformation, (c), (e), (g) with increasing plastic deformation and (i) the final image of the failed specimen in situ as well as j the fracture surface post mortem with an inset showing the depth of the deformed point features. The corresponding strain distributions (b), (d), (f), (h) are depicted to the right of each image, including fitted Gaussian distributions of elastic and total strains, respectively.

With the data analysis adjusted by analysis of the elastic loading regime, image correlation evaluation after the elastic regime is shown in Fig. 7. There, the first image (Fig. 7a) corresponds to the last evaluated frame before plastic deformation, as evident by the narrow single Gaussian distribution of strains along the tensile axis (Fig. 7b). The following frame (Fig. 7c) shows already a slight strain localization and the corresponding distribution (Fig. 7d) depicts a deviation from a purely symmetrical shape. Under the assumption of two strained regions, one still elastically loaded, while the other one already includes plastic deformation, the distribution can be split in two, as show in Fig. 7d, where the one with the lower mean value (1.27 ± 0.12%, orange) corresponds to the elastic regime and the other (3.46 ± 0.34%, green) reflects the total strain in the localized regime. The given uncertainty estimates of the means are calculated from the covariance matrix by the lmfit 1.0.1 package using the python 3.7 programming language. With increasing loading, the strain-magnitude in the localized area increases as depicted in Fig. 7e, and also the strain distribution exhibits a more pronounced split into two regions (Fig. 7f), with the elastic strain regime at 1.69 ± 0.06% and the total strain in the localized regime at 7.19 ± 0.41%. Further increase in loading leads to an even larger area of strain localization (Fig. 7g), with an elastic strain of 2.01 ± 0.11% and a total strain of 11.12 ± 0.80% (Fig. 7h). The strain distribution of the localized part before final failure (Fig. 7h) can be argued to depict multiple peaks with even more nuances in different strain regimes. However, based on the fact that pre-processing of the data was necessary to ensure a smooth strain-field, we are to date not confident that the localized regime should be divided further at this point. The specimen after final failure is depicted in Fig. 7i, where the ductile fracture surface is under approximately 45° inclination with respect to the loading axis. The position and orientation of the strain localization before failure seems in agreement with the arrangement of the fracture surface, suggesting a major contribution of shear stresses and therefore pronounced plasticity in this nanocrystalline HEA specimen.

Furthermore, the fracture surface of the specimen after final failure (Fig. 7j) depicts reduction of the cross-sectional area in both directions perpendicular to the loading direction. Under the assumption of equal reduction in both directions, one can evaluate a cross-sectional area *A*_red_ in the necked volume of the specimen based on the plastic strain in perpendicular direction as:

8 $$A_{{{\text{red}}}} = \left( {1 + \varepsilon_{{yy,{\text{plastic}}}} } \right)^{2} A_{0}$$

where *A*_*0*_ is the unnecked cross-section and *ε*_*yy,*plastic_ is the plastic part of the strain component perpendicular to the loading direction. This plastic strain can be evaluated as difference between the total strain in the strongly necking region and the overall elastic strain, and is estimated based on the difference of the two means in the *ε*_*yy*_ distribution for each frame, in analogy to the distributions shown in Fig. 7 for the *x*-direction.

Furthermore, the inset in Fig. 7j depicts the enlarged red region, where the depth of the point features can be measured, resulting in 185 nm for the shortest and 285 nm for the longest feature. However, as this is the region with the highest plastic deformation it is reasonable to assume that the deformed features are deeper than the initial ones, which suggests these measurements as the upper bound of the feature depth. In conjunction with the depth also the width of the features can increase as evident in Fig. 7i. However, as the proposed methods works on the basis of tracking the maximum of such a feature it can be assumed that the influence thereof is negligible.

### Comparison with commercial software

**Figure 8:**
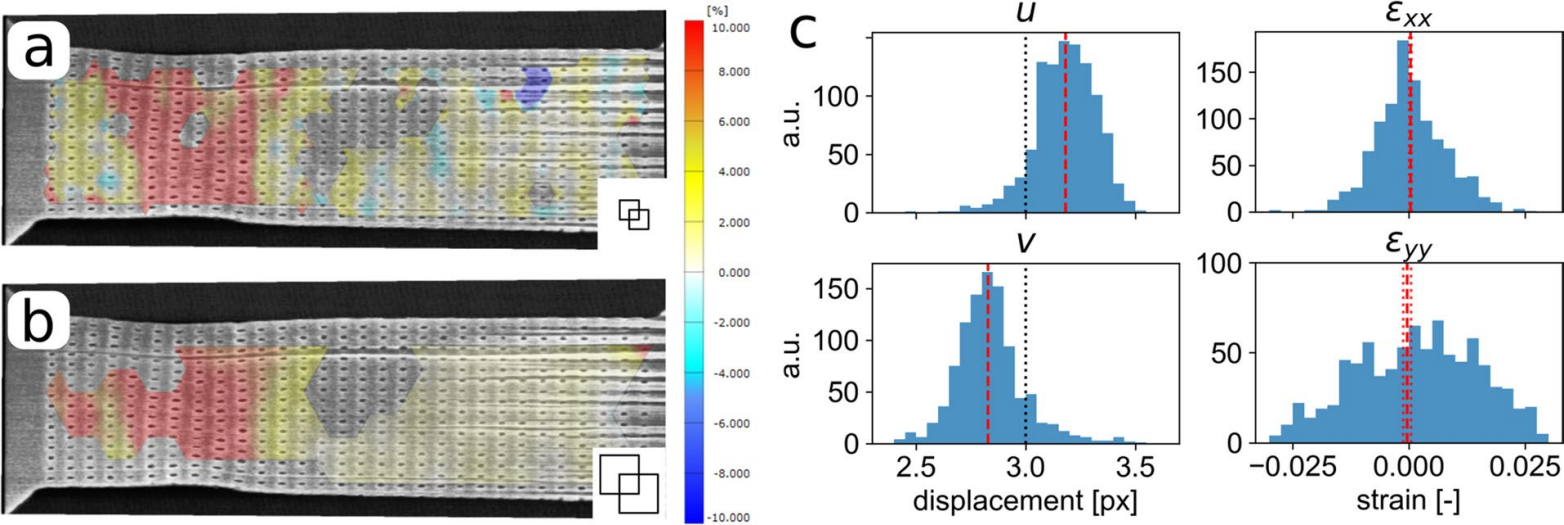
GOM correlate evaluations of the last frame before final failure with (a) 20 px window size and (b) 40 px window size, where the evaluation windows are depicted as overlapping black squares in the bottom left, respectively. (c) Displacement (*u*,*v*) and strain (*ε*_*xx*_,*ε*_*yy*_) distributions of the initial frame manually translated by 3 px in each direction. The dashed red lines depict the distribution means, while the dotted black lines depict the applied 3 px displacement.

To compare the previously shown results with data obtained from commercial DIC software based on classical cross-correlation of grayscale subset windows, the images were evaluated using GOM correlate (GOM GmbH, Braunschweig, Germany) with the suggested standard processing parameters and a window overlap of 30%. In Fig. 8a, b the solutions for the strain along the specimen (*x*-direction) are depicted evaluated at the last frame before final failure, with evaluation window sizes of 20 px and 40 px, respectively. The individual evaluation window sizes and overlaps are depicted in the respective lower left corners as black squares. It is evident that the commercial software is capable of resolving the maximum strain in the range of 10%. In the 20 px case it still shows an evident amount of stochasticity, while in the 40 px case the strain field is much smoother. However, both configurations failed to fully capture the whole strain field on the specimen surface, as for some parts convergence was not achieved, which gives further confirmation that the methodology proposed herein could enhance the evaluation of noisier data, even compared to highly optimized commercial software solutions.

### Resolution of the feature tracking image correlation method

To understand the resolution of the proposed technique, the initial frame was correlated with a copy of itself, which was shifted manually by 3 px in *x*- and *y*-direction. The resulting displacement (*u*,*v*) as well as strain (*ε*_*xx*_,*ε*_*yy*_) -distributions are shown in Fig. 8c, where it is evident that for the given test the displacement is slightly overestimated in *x*-direction (*u*) and slightly underestimated in *y*-direction (*v*). However, the total difference between the mean of the distributions (red dashed line) and the manually translated 3 px (black dotted line) is only 6%, independent of direction. Furthermore, the strain distribution seems to be independent of whether the displacement is over- or underestimated, as the mean values are centred nearly perfectly at a strain of 0 (mean: *ε*_*xx*_ = 0.00034; *ε*_*yy*_ = − 0.00034), which is to be expected in a pure translation.

### DIC based stress strain data in comparison to spherical nanoindentation

**Figure 9:**
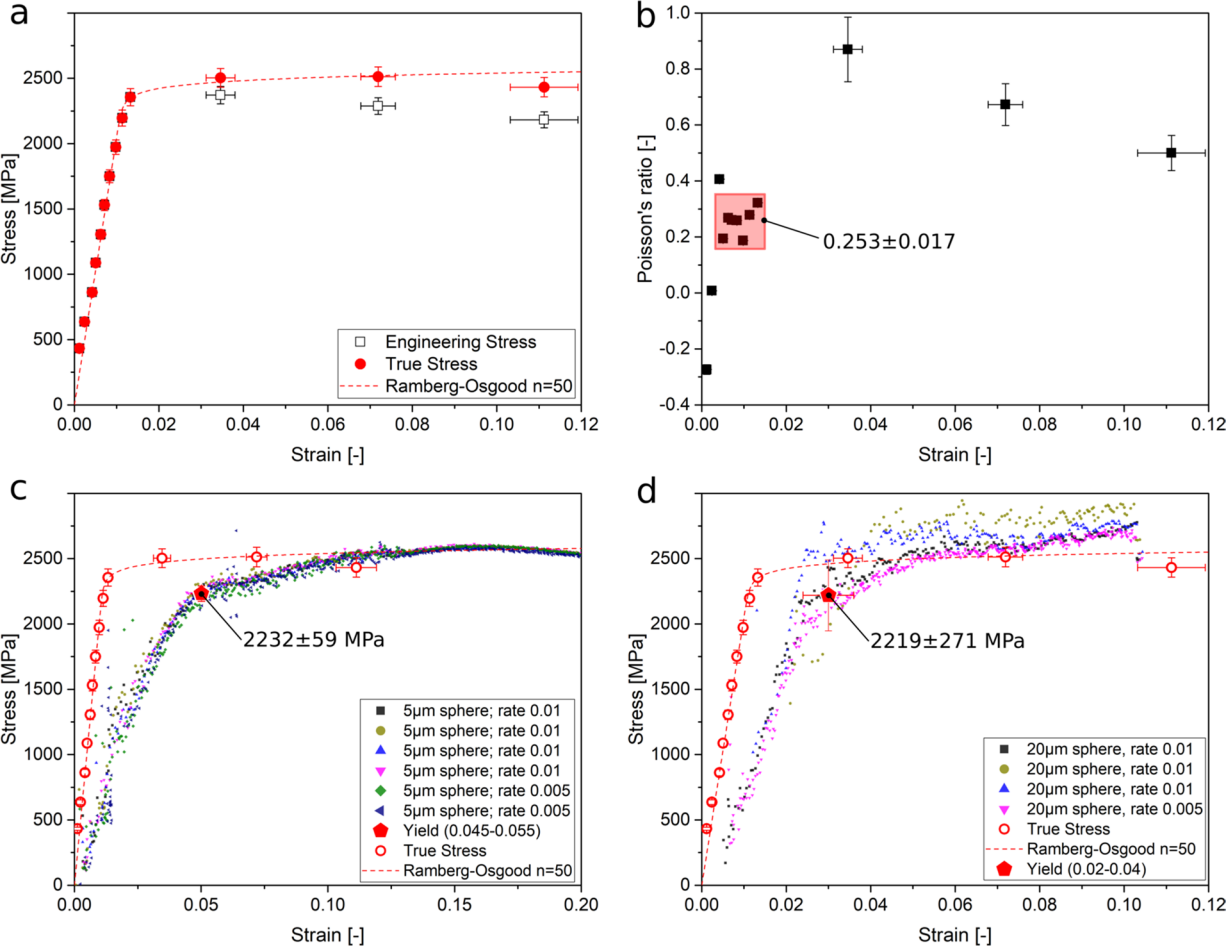
(a) Stress–strain data for the nanocrystalline Cantor alloy specimen as engineering stress (black open squares) and corrected true stress (red filled circles), evaluated using the total strain from the presented image correlation technique. For comparison, a Ramberg–Osgood type relation with *n* = 50 is shown as dotted red curve. (b) The Poisson’s ratio for all evaluated points with the red transparent region depicting the physically reasonable values used for averaging. True stress–strain data in comparison with stress–strain data from spherical nanoindentation with radii of (c) 5 µm and (d) 20 µm.

The resulting tensile stress–strain data is shown in Fig. 9a, where the black open squares depict the engineering stress–strain data and the red filled circles reflect the data corrected by reduced cross-sectional area (Eq. 8), reflecting the true physical specimen stress. Error estimates are based on the standard error of the mean of the respective distributions for the strain as well as uncertainty propagation of uncorrelated input quantities, with a measured load noise level of 50 µN and an assumed geometry measurement error of ± 3 px. It is evident that the engineering stress–strain data shows nearly instant softening after the elastic regime, which can be addressed to purely geometrical shape change, whereas the corrected stress–strain data depicts a flat flow-stress plateau with only very slight actual softening for the last data point, a flow characteristic well known for nanocrystalline materials [32, 33]. The classical 0.2% offset of the elastic slope for the evaluation of the yield onset intersects almost perfectly at the last point before major plastic deformation is evident in the strain fields and distributions, resulting in a yield stress of *σ*_*Y* _= 2355 ± 66 MPa. Furthermore, to describe the stress–strain behaviour of a strain hardening material continuously, one can utilize the well-known Ramberg–Osgood type relation, as [34]:

9 $$\varepsilon = \frac{\sigma }{E} + 0.002\left( {\frac{\sigma }{{\sigma_{Y} }}} \right)^{n}$$

where *n* is a hardening parameter that approaches infinity for perfect elastic–plastic transitions. With the yield strength and elastic modulus specified, one can calculate the mean-square stress error between model and true physical stress data. This was done manually for *n* = 30, *n* = 50 and *n* = 100, respectively, and showed qualitatively only little difference, with *n* = 50 giving the lowest mean-square error. However, as the specimen shows nearly no hardening in the beginning and softening at the end, and because the data in the plastic regime is rather sparse, the hardening exponent should be considered as a qualitative measure only.

Furthermore, evaluating the total strain field allows for an estimation of Poisson’s ratio *ν* as:

10 $$\nu = - \frac{{\varepsilon_{yy} }}{{\varepsilon_{xx} }}$$

where *ε*_*xx*_ and *ε*_*yy*_ can be taken as the total strains parallel and perpendicular to the loading axis, respectively. The results are shown in Fig. 9b, where the initial three values in the elastic regime (− 0.27, 0.01 and 0.41) can be considered non-physical, as the difference between them is very distinct and the values are outside the quantities for common metallic materials (~ 0.2–0.35). This strong deviation in the early loading steps is most likely a result of the low absolute displacements, and therefore, relatively reduced resolution. The physically sound data is indicated in the red box in Fig. 9b, where the average Poisson’s ratio equates to *ν* = 0.253 ± 0.017, which is in excellent agreement with data measured by resonance peak experiments on bulk material (*ν* = 0.25) [30]. Furthermore, it is evident that the data in the plastic regime overestimates the maximum of *ν* = 0.5 for full plasticity quite significantly. A reason for that could be that the impending shear localization influences the distribution in perpendicular direction and therefore the peak maximum is shifted towards higher values. However, as the Poisson’s ratio is commonly only determined in the elastic regime as a material constant, the data in the plastic regime will be neglected herein.

For comparison, the true-stress strain data (open red circles) is depicted with stress–strain data from spherical nanoindentation experiments (filled symbols) in Fig. 9c, d. The data from the 5 µm sphere (Fig. 9c) shows very reproducible behaviour without distinct influence on varying strain rate from 0.01 to 0.005. The initial elastic regime shows a lower slope and a slight non-linear behaviour from the beginning. A distinct yield onset is evident as the slope changes distinctly at around a strain of 0.05. Evaluating the mean and standard deviation of all data points between 0.045 and 0.055 strain results in a yield stress of 2232 ± 59 MPa, which is around 5% lower than the yield stress evaluated in the tensile experiment. However, the final flow stress level is in excellent agreement with the flow stress in the plastic regime of the tensile specimen. The data from the 20 µm sphere depicts an overall higher scatter and higher flow stress, while the elastic regime is slightly steeper and more linear than the data from the 5 µm sphere. The yield onset is not defined as precisely as for the 5 µm sphere, but evaluating again the average and standard deviation of all data points between 0.02 and 0.04 results in 2219 ± 271 MPa, which is good agreement with the 5 µm sphere yield onset.

## Discussion

While in situ micro-tensile tests inside the SEM are a rather promising technique to study material properties of components or individual features that are otherwise difficult to address, *e.g.* single crystals, individual precipitates or grain boundaries, it is still rarely utilized due to the challenging experimental conditions. However, in comparison to the frequently employed push-to-pull device method for tensile testing of individual manipulated, placed and fixed objects [8–12], the benefit of having multiple specimens besides each other is non-negligible [17]. While the easier experimental setup of in situ micropillar compression also allows for multiple specimens in one session, it suffers from the fact that due to the constraints from the tip and the base, the observed deformation behaviour is not always exclusively material-dependent [35, 36]. More importantly, the failure behaviour under compression cannot be directly compared to the one under tension. This places in situ microtensile experiments in a unique position, as it still offers the chance for a better statistical testing, while the geometry constraints can be minimized with proper specimen design and deformation to failure can be probed. However, as shown in Fig. 3, the suitable data evaluation is not always straightforward, specifically for high strength materials, *e.g.* nanocrystalline metals. Therefore, utilizing the additional information gained from the images recorded during in situ testing can make a significant impact. While the classical DIC methods have been applied to micron sized specimens and allow for better strain resolution [37, 38], it raises the need for higher-resolution imaging data and is therefore, not suitable for quasi-continuous testing, which is the pre-requisite for various mechanical testing methods due to occurrence of material dependent strain rate sensitive characteristics [39–42]. Furthermore, high plastic deformation has always been a challenge for classical DIC due the fact that the features could deform quite drastically and move a large distance, which is demanding for any greyscale histogram cross-correlation algorithm. Point tracking methods exchanges the high strain resolution in the elastic regime for the possibility to assess large plastic strains and robust evaluation [26, 27]. Commonly, point features are created by remodelling of thin metallic layers [43] resulting in a random structure, or via deposition of metallic constituents [44], e.g. Pt utilizing FIB or electron beam deposition [26, 27, 37]. However, while the use of such methods can be favourable when studying defect driven phenomena that are influenced by the surface structure, it is also more challenging to achieve small and reproducible high-contrast features on the microsamples without initial parameter studies. On the other hand, the use of FIB processed features is straight forward during specimen preparation and avoids the need for additional deposition systems at the cost of geometrical surface structuring and ion implantation. However, since the feature size (Fig. 7j) as well as the implantation depth [45] are only in the range of individual grain dimensions in the given experiment, it is reasonable to assume only minor influence thereof, as the cross-section of the specimen contains multiple thousands of grains. Furthermore, the suggested feature creation method could be scaled in both directions, considering recent developments in femtosecond laser ablation [46, 47] for larger or helium ion microscopy [48] for smaller structures. A further benefit of the method shown herein is the symmetrically structured grid, as it enables a convenient qualitative determination of deformation, even without any image processing, which is hardly evident in random structures. This allows a feasibility check during the in situ experiments and provides intuition regarding the deformation before any evaluation is attempted, which can help with understanding the characteristics of more complex strain fields, e.g. the deformation around a notch.

The comparison of the evaluated stress–strain data based on the presented image correlation technique with spherical-nanoindentation suggests excellent agreement in the plastic flow regime. The 5 µm sphere data is nearly perfectly reproducible, but shows a more transient transition from the elastic to the plastic regime, which makes the evaluation of an elastic modulus as well as a yield onset quite challenging. The 20 µm data shows a more pronounced elastic slope, but a distinct scatter in yield onset and further flow stress level, which can be explained when considering that a flatter shape (20 µm) distributes contact points originating from surface roughness over a larger area in comparison to a shape with higher curvature (5 µm). Consequently, local contact features can introduce a relatively higher stress, which can lead to earlier microyielding [49, 50] in the 5 µm sphere experiments, while the higher contact area in the 20 µm sphere experiments allow for a pronounced elastic contact followed by the stochastics of dislocation nucleation. However, considering the overall agreement of yield onset as well as flow stress level in the plastic regime, the nanoindentation data allows for a confident verification of the yield and flow levels in the tensile experiments achieved by the novel DIC approach. Taking this one step further, with the recent advances in machine learning techniques, one could utilize the presented accurate micro-tensile experiment as high-fidelity data, while the easier accessible spherical nanoindention data could act as high throughput low-fidelity data for plastic properties in conjunction with classical Berkovich indentation for elastic properties to determine the precise stress–strain behaviour for material systems that are otherwise very challenging to address, *e.g.* thin films or precipitates.

## Summary and conclusion

An in situ micro-tensile experiment on a high strength nanocrystalline HEA is conducted with additional DIC based feature tracking to enable local strain evaluation on the specimen. The surface features were produced through FIB milling and acted as corner points for quadrilateral elements to calculate a Green–Lagrange strain after finite strain theory, which takes into account large plastic deformation. The calculated strain was further evaluated by statistical means. Thus, our approach facilitates the measurement of an actual strain on the specimen surface without compliance issues, and takes into account the cross-sectional area reduction to correct for geometrical softening to assess the true stress instead of the engineering stress only. Furthermore, the full two-dimensional strain field enables an evaluation of Poisson’s ratio, which has not been measurable before, while the overall flow level in the plastic regime was verified by means of spherical nanoindentation. Taken together, this approach allows for precise rate controlled uniaxial mechanical testing of high strength materials within the present micro-tensile testing setup. Furthermore, the presented method could serve as a basis for the evaluation of micron-sized specimens of various geometries, *e.g.* pillar compression, cantilever bending or for evaluation of distinct local features, *e.g.* indented or notched regions.

## Materials and methods

### Material and sample preparation

The material used in this study is an equiatomic five-component CoCrFeMnNi high entropy alloy (HEA), commonly known as the Cantor alloy [51], which was processed by high pressure torsion (HPT) as described by Schuh et al. [52], to achieve a nanocrystalline microstructure with grain size in the order of 50 nm. A wedge shaped specimen was prepared from the HPT disk by grinding and polishing to a final thickness of ~ 20 µm. Thereafter, a dogbone shaped microtensile specimen with 45° angles (Fig. 2) was prepared by focussed ion beam milling (FIB, LEO 1540XB, Carl Zeiss AG, Oberkochen, Germany) with 30 kV acceleration voltage and a current of 10 nA. All 90° angles of the specimen were fabricated with overtilting to counteract any FIB induces tapers. In multiple passes the current was reduced to 100 pA for the final polishing steps [53]. Thereafter, a point pattern was produced perpendicular to the top surface of the tensile specimen, by using a dotted bitmap mask in conjunction with the built in feature-milling software of the FIB, with a current of 50 pA, without any additional need for metal deposition or alike.

### Micro-tensile testing

The specimen was tested in situ in an SEM (DSM982, Carl Zeiss AG, Oberkochen, Germany) utilizing an UNAT-SEM 1 microindentation device (ASMEC Gmbh, Dresden, Germany) as described by Kiener et al*.* [6, 54]. The gripper was fabricated from a polycrystalline W needle (usually used in micromanipulators) with an open gripping width of 9.8 µm via FIB machining. The experiment was conducted in displacement controlled mode with a loading rate of 20 nm/s, which corresponds to a nominal engineering strain rate of 10^–3^ s^−1^. To assure a quasi-continuous acquisition of images during the experiment, the SEM was adjusted to a scan speed requiring 660 ms per frame, which was found to result in a good trade-off between image quality and temporal resolution.

### Spherical nanoindentation

Spherical nanoindentation was conducted and evaluated following the methodology after Leitner et al*.* [55] using a G200 Nanoindenter (KLA Corporation, Milpitas, California, USA) equipped with spherical diamond tips obtained from Synton MDP (Nidau, Switzerland). The tests were conducted using the continuous stiffness measurement protocol with a frequency of 45 Hz and a displacement amplitude of 2 nm. To address potential systematic influences, spheres with radii of 5 µm and 20 µm were used and the strain rate was varied between 0.01 s^−1^ and 0.005 s^−1^.
